# Gene expression profiling of trout muscle during flesh quality recovery following spawning

**DOI:** 10.1186/s12864-021-08228-3

**Published:** 2022-01-04

**Authors:** Yéléhi-Diane Ahongo, Aurélie Le Cam, Jérôme Montfort, Jérôme Bugeon, Florence Lefèvre, Pierre-Yves Rescan

**Affiliations:** INRAE, UR 1037, LPGP Fish Physiology and Genomics, Campus de Beaulieu, F-35042 Rennes, France

**Keywords:** Salmonids, Post-spawning evolution, Flesh quality, Muscle, Transcriptome

## Abstract

**Background:**

Sexual maturation causes loss of fish muscle mass and deterioration of fillet quality attributes that prevent market success. We recently showed that fillet yield and flesh quality recover in female trout after spawning. To gain insight into the molecular mechanisms regulating flesh quality recovery, we used an Agilent-based microarray platform to conduct a large-scale time course analysis of gene expression in female trout white muscle from spawning to 33 weeks post-spawning.

**Results:**

In sharp contrast to the situation at spawning, muscle transcriptome of female trout at 33 weeks after spawning was highly similar to that of female trout of the same cohort that did not spawn, which is consistent with the post-spawning flesh quality recovery. Large-scale time course analysis of gene expression in trout muscle during flesh quality recovery following spawning led to the identification of approximately 3340 unique differentially expressed genes that segregated into four major clusters with distinct temporal expression profiles and functional categories. The first cluster contained approximately 1350 genes with high expression at spawning and downregulation after spawning and was enriched with genes linked to mitochondrial ATP synthesis, fatty acid catabolism and proteolysis. A second cluster of approximately 540 genes with transient upregulation 2 to 8 weeks after spawning was enriched with genes involved in transcription, RNA processing, translation, ribosome biogenesis and protein folding. A third cluster containing approximately 300 genes upregulated 4 to 13 weeks after spawning was enriched with genes encoding ribosomal subunits or regulating protein folding. Finally, a fourth cluster that contained approximately 940 genes with upregulation 8 to 24 weeks after spawning, was dominated by genes encoding myofibrillar proteins and extracellular matrix components and genes involved in glycolysis.

**Conclusion:**

Overall, our study indicates that white muscle tissue restoration and flesh quality recovery after spawning are associated with transcriptional changes promoting anaerobic ATP production, muscle fibre hypertrophic growth and extracellular matrix remodelling. The generation of the first database of genes associated with post-spawning muscle recovery may provide insights into the molecular and cellular mechanisms controlling muscle yield and fillet quality in fish and provide a useful list of potential genetic markers for these traits.

**Supplementary Information:**

The online version contains supplementary material available at 10.1186/s12864-021-08228-3.

## Background

As a result of the increasing demand for fish consumption, aquaculture has become the type of animal food production with the fastest growth in recent decades [1]. Muscle growth and fillet quality are important traits that impact the profitability of the fish breeding industry. However, flesh qualities are not constant throughout the fish lifecycle. For example, fertile diploid female trout particularly those that are farmed for egg production, exhibit low flesh quality and a decrease in fillet yield around the spawning period and are thus not suitable for the market. Flesh deterioration at spawning is due in large part to muscle atrophy associated with protein catabolism which is exacerbated in the muscle tissue of maturing female trout to provide the energy and nutrients necessary for egg development [2]. In addition, some of the lipids mobilized during sexual maturation originate from muscle store [3–5].

Several transcriptomic analyses have been performed to decipher the mechanisms underlying muscle changes observed during the sexual maturation. An initial microarray gene expression study showed that sexual maturation-induced atrophy of axial muscle in gravid trout compared to sterile trout was associated with (i) upregulation of genes involved in catheptic and collagenase proteolytic pathways and genes involved in mitochondrial aerobic ATP production and (ii) downregulation of genes regulating RNA processing and protein biosynthesis and genes encoding myofibrillar and extracellular matrix proteins [6]. Further studies using RNA-Seq techniques have essentially confirmed these data and revealed, in the same model, increased expression of many genes encoding components of the muscle “degradome” particularly those forming the ubiquitin proteasome system, and decreased expression of genes involved in amino acid and fat biosynthesis [7, 8]. Additionally, the expression of β-oxidation genes in muscle has been reported to be higher in fertile than in sterile (triploid) trout, suggesting that fatty acid mobilisation within muscle is enhanced during sexual maturation [9]. Consistent with the transcriptomic data, the proteomic signature of muscle atrophy in fertile fish compared to sterile (triploid) female trout shows decreased abundance of enzymes involved in anaerobic respiration and protein biosynthesis [10].

We recently showed that sexual maturation-associated deterioration in flesh quality can be reversed in trout post spawning. Notably, we observed an increase in fillet yield during the post spawning period, suggesting an accretion of protein mass in muscle fibres and a significant increase in intramuscular fat content [11]. Restoring trout muscle quality after spawning is of particular interest for aquaculture profitability and sustainability and deserves specific investigation. Currently, very little is known regarding the molecular mechanisms regulating flesh quality restoration after spawning. In this study, we used microarray technology to explore the temporal changes in muscle gene expression following spawning and to infer the molecular pathways associated with post-spawning flesh quality recovery. Additionaly, to further characterize the specificity of the muscle molecular signature following spawning, we compared this signature with that of hyperplastic growth zones of the late trout embryo [12] and that reported during a fasting/refeeding schedule [13].

## Results

To gain insight into the transcriptomic changes associated with flesh quality restoration after spawning we performed microarray hybridisations using RNA extracted from the muscle tissues of mature female trout sampled at 0, 2, 4, 8, 13, 16, 24, and 33 weeks after spawning (PS0, PS2, PS4, PS8, PS13, PS16, PS24, and PS33) and from the muscle tissues of immature (control) female trout. Immature (control) female trout were sampled at the beginning (C0) and at the end (C33) of the experiment and belonged to the same cohort as trout that experienced spawning.

### Comparison of muscle transcriptome in mature and immature (control) trout at spawning time and 33 weeks after spawning time

Using an unpaired *t* test (BH corrected *p*-val < 0.05), we first compared the muscle transcriptomes in mature and in immature (control) trout at the beginning and end of the experiment. We found that the muscle transcriptome of trout that had just spawned (PS0) was clearly different from that of control trout (C0), as revealed by the identification of approximately 4700 unique DEGs between PS0 and C0. In contrast, the trout muscle transcriptome at 33 weeks post-spawning (PS33) was virtually the same as that found in the 33 weeks control (C33), as indicated by the lack of identification of any DEGs between PS33 and C33 via unpaired *t* test. This shows that the trout muscle transcriptome after spawning evolved to eventually become similar to that of trout that did not spawn, which is consistent with the post-spawning muscle restoration and flesh quality recovery we recently reported [11].

### Temporal gene expression profiling after spawning: an overview

**Fig. 1:**
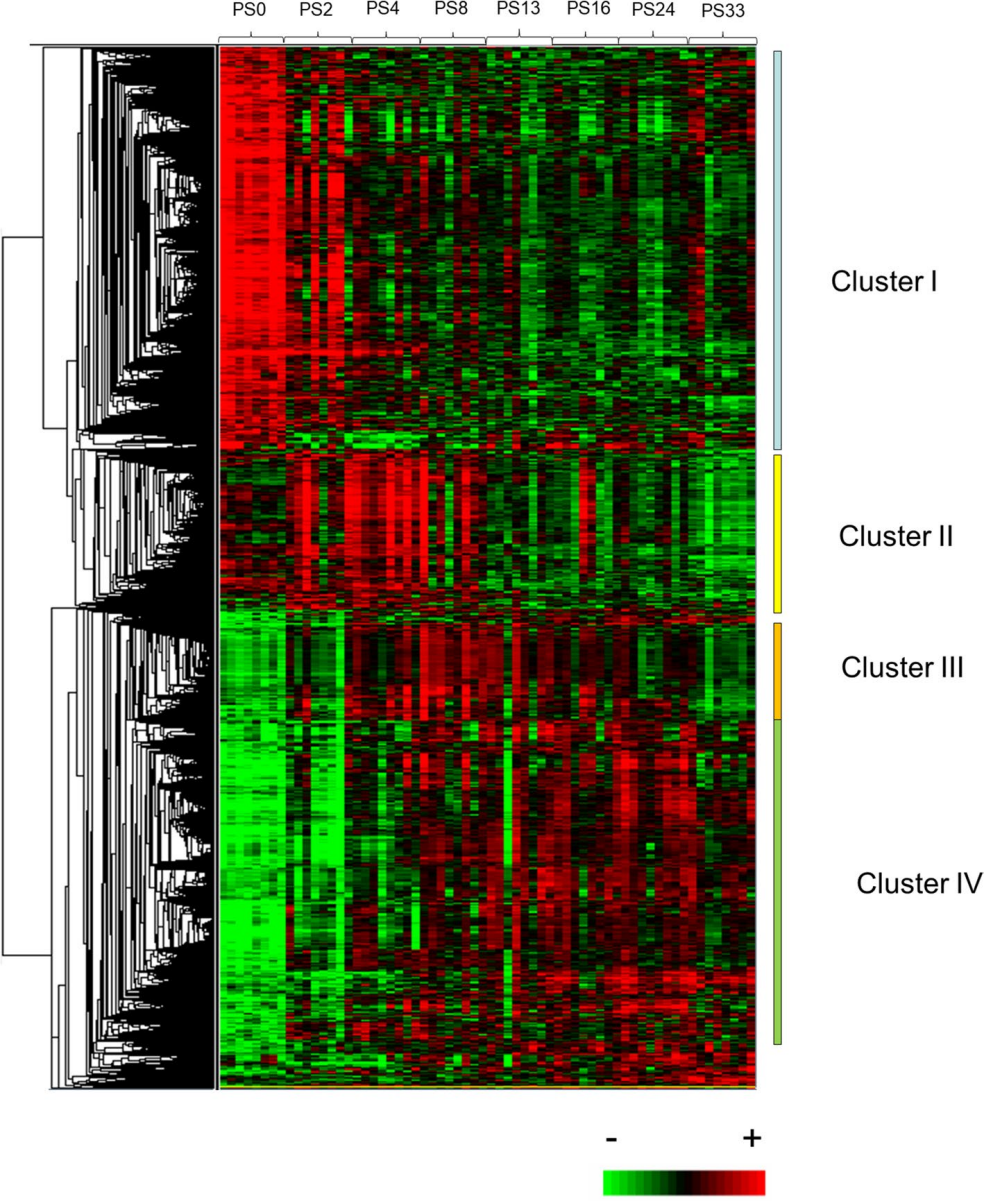
Heat map of the supervised hierarchical clustering of differentially expressed genes in trout muscle during the post-spawning period. The clustering of differentially expressed genes led to the formation of four distinct clusters (I, II, II and IV). Each row represents the temporal expression pattern of a single gene and each column corresponds to a single sample. Columns 1 to 8: PS0 = trout muscle at spawning. Columns 9 to 16: PS2 = trout muscle 2 weeks after spawning. Columns 17 to 24: PS4 = trout muscle 4 weeks after spawning. Columns 25 to 32: PS8 = trout muscle 8 weeks after spawning. Columns 33 to 40: PS13 = trout muscle 13 weeks after spawning. Columns 41 to 48: PS16 = trout muscle 16 weeks after spawning. Columns 49 to 56: PS24 = trout muscle 24 weeks after spawning. Columns 57 to 64: PS33 = trout muscle 33 weeks after spawning. Expression levels are represented by a colour tag, with red representing the highest levels and green the lowest levels of expression

Next, we aimed to characterize the changes in the female trout muscle transcriptome associated with flesh quality recovery following spawning. For this purpose, an ANOVA (Benjamini-Hochberg method with a FDR < 0.05) and a fold change > 3 were used as criteria for defining genes whose expression levels were significantly different across all the stage of sampling (0, 2, 4, 8, 13, 16, 24 and 33 weeks after spawning). This analysis led to the identification of approximately 3340 unique differentially expressed genes (DEGs). Hierarchical clustering of DEGs resulted in the formation of four major gene clusters (clusters I-IV) (Fig. 1, see also Additional file 1 showing mean expression curves across time points for all the genes contained in clusters I-IV). We found that cluster I contained approximately 1350 genes with peak expression at spawning and downregulation after spawning. Cluster II included approximately 540 genes with transient upregulation between 2 and 8 weeks after spawning. Cluster III comprised approximately 300 genes upregulated 4 to 13 weeks after spawning, and cluster IV contained approximately 940 genes whose expression level progressively increased from 8 to 24 weeks after spawning.

### Cluster I: genes with peak expression at spawning and downregulation after spawning

Cluster I comprised approximately 1350 unique genes highly expressed in the muscle tissues of trout that had just spawned and downregulated after spawning. DAVID analysis of genes belonging to cluster I and annotated with ontology identifiers showed enrichment for GO terms linked to mitochondrial oxidative phosphorylation, the fatty acid catabolic process, and the tricarboxylic acid cycle. Cluster I was also highly enriched with genes involved in proteolysis. Consistent with the enrichment of this GO term, we found the classical markers of muscle atrophy Murf1/Trim63 and Atrogin/FBXO32/Mafbx, as well as many genes regulating the proteasomal ubiquitin-dependent protein catabolic process, such as genes encoding subunits of the proteasome 26S complex (16 upregulated genes). In addition, GO terms linked to proteolysis were enriched for genes regulating lysosome activity and genes participating in autophagy (for details see Tables 1, 2 and 3; the list of genes that formed major functional categories of cluster I are in Additional file 2).

**Table 1:** Functional categories inferred from genes with pick expression at spawning and downregulation after spawning (cluster I), upregulated 2 to 8 weeks after spawning (cluster II), upregulated 4 to 13 weeks after spawning (cluster III) and upregulated 8 to 24 weeks after spawning (cluster IV). *P*-Value represents the EASE score (modified Fisher exact *P*-Value) of a GO generated by DAVID tool after GO enrichment analysis

	KEGG Pathway	Count	*P*-Value	GO Cell component	Count	*P*-value	GO biological process	Count	*P*-value
**Cluster I**	Proteasome	**31**	1.4E-24	Mitochondrion	**121**	1.0E-19	Tricarboxylic acid cycle	**13**	6.6E-10
	Oxidative phosphorylation	**38**	7.4E-13	Mitochondrial inner membrane	**35**	3.1E-10	Proteasome-mediated ubiquitin-dependent protein catabolic process	**26**	7.0E-9
	Citrate cycle	**15**	1.4E-8	Mitochondrial respiratory chain complex 1	**15**	E4.5E-7	Fatty acid beta-oxidation using acyl-CoA dehydrogenase	**11**	5.5E-9
	Fatty acid degradation	**16**	7.6E-8	Proteasome complex	**11**	6.0E-7	Autophagosome assembly	**15**	3.4E-8
	Lysosome	**22**	2.1E-4	Peroxysome	**13**	1.1E-4	Autophagy	**9**	2.5E-4
				Autophagosome	**9**	9.9E-4			
**Cluster II**	Ribosome biogenesis in eucaryote	**32**	1.0E-29	Nucleolus	**77**	8.2E-35	rRNA processing	**18**	2.6E-16
	RNA polymerase	**9**	6.5E-7	Small-subunit processome	**17**	1.5E-19	Translation	**23**	4.2E-9
	Spliceosome	**16**	2.1E-6	Preribosome, large subunit precursor	**13**	3.3E-13	Protein folding	**10**	4.4E-4
	RNA transport	**16**	8.7E-5	Intracellular ribonucleoprotein complex	**14**	2.0E-9			
				Mitochondrion	**44**	3.9E-6			
**Cluster III**	Ribosome	**62**	3.0E-70	Cytosolic large ribosomal subunit	**34**	1.0E-10	Protein folding	**8**	2.9E-4
				Cytosolic small ribosomal subunit	**21**	1.4E-29			
				nucleolus	**28**	3.1E-29			
				chaperonin-containing T-complex	**6**	8.3E-9			
**Cluster IV**	Glycolysis/neoglucogenesis	**24**	5.9E-16	Proteinaceous extracellular matrix	**26**	6.3E-8	Glycolytic process	**15**	1.9E-13
	Biosynthesis of amino acids	**22**	1.4E-12	Collagen trimer	**14**	3.3E-7	Collagen fibril organisation	**10**	2.3E-6
	ECM-receptor interaction	14	1.0E-4	basement membrane	**12**	3.1E-5	Muscle contraction	**14**	7.5E-6
				Troponin complex	**5**	1.6E-4	Cell proliferation	**16**	1.5E-3
				Myosin complex	**10**	2.0E-4	Regulation of spindle microtubule to kinetochore	**4**	1.5E-3
							Cell division	**17**	5.5E-3

**Table 2:** Genes with pick expression at spawning and downregulation after spawning (cluster I) and involved in proteasome

Genes from cluster I involved in proteasome	
proteasome 26S subunit, ATPase 1 (PSMC1)	
proteasome 26S subunit, ATPase 2 (PSMC2)	
proteasome 26S subunit, ATPase 3 (PSMC3)	
proteasome 26S subunit, ATPase 4 (PSMC4)	
proteasome 26S subunit, ATPase 6 (PSMC6)	
proteasome 26S subunit, non-ATPase 1 (PSMD1)	
proteasome 26S subunit, non-ATPase 11 (PSMD11)	
proteasome 26S subunit, non-ATPase 12 (PSMD12)	
proteasome 26S subunit, non-ATPase 13 (PSMD13)	
proteasome 26S subunit, non-ATPase 14 (PSMD14)	
proteasome 26S subunit, non-ATPase 2 (PSMD2)	
proteasome 26S subunit, non-ATPase 3 (PSMD3)	
proteasome 26S subunit, non-ATPase 4 (PSMD4)	
proteasome 26S subunit, non-ATPase 6 (PSMD6)	
proteasome 26S subunit, non-ATPase 7(PSMD7)	
proteasome 26S subunit, non-ATPase 8 (PSMD8)	
proteasome activator subunit 4 (PSME4)	
proteasome maturation protein (POMP)	
proteasome subunit alpha 1 (PSMA1)	
proteasome subunit alpha 3 (PSMA3)	
proteasome subunit alpha 4 (PSMA4)	
proteasome subunit alpha 5 (PSMA5)	
proteasome subunit alpha 6 (PSMA6)	
proteasome subunit alpha 7 (PSMA7)	
proteasome subunit alpha 8 (PSMA8)	
proteasome subunit beta 1 (PSMB1)	
proteasome subunit beta 2 (PSMB2)	
proteasome subunit beta 4 (PSMB4)	
proteasome subunit beta 5 (PSMB5)	
proteasome subunit beta 7 (PSMB7)	
split hand/foot malformation type 1 (SHFM1)

**Table 3:** Genes with pick expression at spawning and downregulation after spawning (cluster I) and involved in oxidative phosphorylation

Genes from cluster I involved in oxidative phosphorylation	
ATP synthase, H+ transporting, mitochondrial F1 complex, beta polypeptide (ATP5B)	
ATP synthase, H+ transporting, mitochondrial Fo complex subunit B1 (ATP5F1)	
ATP synthase, H+ transporting, mitochondrial Fo complex subunit C3 (subunit 9) (ATP5G3)	
ATP synthase, H+ transporting, mitochondrial Fo complex subunit E (ATP5I)	
ATPase H+ transporting V0 subunit a1 (ATP6V0A1)	
ATPase H+ transporting V0 subunit e2 (ATP6V0E2)	
ATPase H+ transporting V1 subunit A (ATP6V1A)	
ATPase H+ transporting V1 subunit G1 (ATP6V1G1)	
COX10 heme A:farnesyltransferase cytochrome c oxidase assembly factor (COX10)	
NADH:ubiquinone oxidoreductase core subunit S1 (NDUFS1)	
NADH:ubiquinone oxidoreductase core subunit S7 (NDUFS7)	
NADH:ubiquinone oxidoreductase core subunit S8 (NDUFS8)	
NADH:ubiquinone oxidoreductase core subunit V1 (NDUFV1)	
NADH:ubiquinone oxidoreductase subunit A10 (NDUFA10)	
NADH:ubiquinone oxidoreductase subunit A12 (NDUFA12)	
NADH:ubiquinone oxidoreductase subunit A5 (NDUFA5)	
NADH:ubiquinone oxidoreductase subunit A6 (NDUFA6)	
NADH:ubiquinone oxidoreductase subunit A9 (NDUFA9)	
NADH:ubiquinone oxidoreductase subunit B10 (NDUFB10)	
NADH:ubiquinone oxidoreductase subunit B3 (NDUFB3)	
NADH:ubiquinone oxidoreductase subunit B4 (NDUFB4)	
NADH:ubiquinone oxidoreductase subunit B5 (NDUFB5)	
NADH:ubiquinone oxidoreductase subunit B6 (NDUFB6)	
NADH:ubiquinone oxidoreductase subunit B7 (NDUFB7)	
cytochrome b-c1 complex subunit 9 (UQCR10)	
cytochrome c oxidase subunit 4I1 (COX4I1)	
cytochrome c oxidase subunit 5A (COX5A)	
cytochrome c oxidase subunit 7A2 like (COX7A2L)	
cytochrome c oxidase subunit 7B (COX7B)	
cytochrome c oxidase subunit VIIa polypeptide 2 (liver) (COX7A2)	
cytochrome c1 (CYC1)	
succinate dehydrogenase complex flavoprotein subunit A (SDHA)	
succinate dehydrogenase complex iron sulfur subunit B (SDHB)	
succinate dehydrogenase complex subunit C (SDHC)	
ubiquinol-cytochrome c reductase complex III subunit VII (UQCRQ)	
ubiquinol-cytochrome c reductase core protein I (UQCRC1)	
ubiquinol-cytochrome c reductase core protein II (UQCRC2)	
ubiquinol-cytochrome c reductase, Rieske iron-sulfur polypeptide 1 (UQCRFS1)	

### Cluster II: genes upregulated 2 to 8 weeks after spawning

Cluster II contained approximately 540 unique genes. DAVID analysis of the eligible genes composing cluster II revealed enrichment for GO terms linked to transcription, RNA splicing and ribonucleoprotein complex biogenesis. Cluster II was enriched with genes involved in translation (including notably 8 translation initiation factors), ribosome biogenesis (which determines translation capacity) and protein folding. Cluster II was also enriched with genes encoding components of mitochondria, such as genes encoding mitochondrial ribosomes. However, in contrast to cluster I, cluster II did not include genes involved in mitochondrial oxidative phosphorylation. Overall, cluster II was dominated by genes involved in cellular biosynthetic processes necessary for protein mass accretion (for details see Tables 1, 4 and 5; the list of genes that formed the major functional categories of cluster II are in Additional file 3).

**Table 4:** Genes upregulated 2 to 8 weeks after spawning (cluster II) and involved in ribosome biogenesis

Genes from cluster II involved in ribosome biogenesis	
5′-3′ exoribonuclease 2 (XRN2)	
BMS1, ribosome biogenesis factor (BMS1)	
FCF1 rRNA-processing protein (FCF1)	
G protein nucleolar 3 like (GNL3L)	
G protein nucleolar 3 (GNL3)	
GAR1 ribonucleoprotein (GAR1)	
HEAT repeat containing 1 (HEATR1)	
IMP3, U3 small nucleolar ribonucleoprotein (IMP3)	
IMP4 homolog, U3 small nucleolar ribonucleoprotein (IMP4)	
M-phase phosphoprotein 10 (MPHOSPH10)	
N-acetyltransferase 10 (NAT10)	
NHP2 ribonucleoprotein (NHP2)	
NIN1/PSMD8 binding protein 1 homolog (NOB1)	
NMD3 ribosome export adaptor (NMD3)	
NOP10 ribonucleoprotein (NOP10)	
NOP56 ribonucleoprotein (NOP56)	
NOP58 ribonucleoprotein (NOP58)	
POP1 homolog, ribonuclease P/MRP subunit (POP1)	
PWP2 periodic tryptophan protein homolog (yeast) (PWP2)	
RNA exonuclease 2 (REXO2)	
RNA terminal phosphate cyclase like 1 (RCL1)	
UTP14A small subunit processome component (UTP14A)	
UTP18, small subunit processome component (UTP18)	
UTP6, small subunit processome component (UTP6)	
WD repeat domain 3 (WDR3)	
WD repeat domain 36 (WDR36)	
WD repeat domain 43 (WDR43)	
casein kinase 2 alpha 1 (CSNK2A1)	
dyskerin pseudouridine synthase 1 (DKC1)	
eukaryotic translation initiation factor 6 (EIF6)	
fibrillarin (FBL)	
ribonuclease P/MRP subunit p38 (RPP38)

**Table 5:** Genes upregulated 2 to 8 weeks after spawning (cluster II) and involved in transcription and spliceosome

Genes from cluster II involved in transcription or spliceosome	
RNA polymerase I subunit A (POLR1A)	
RNA polymerase I subunit B (POLR1B)	
RNA polymerase I subunit C (POLR1C)	
RNA polymerase I subunit E (POLR1E)	
RNA polymerase II subunit E (POLR2E)	
RNA polymerase III subunit B (POLR3B)	
RNA polymerase III subunit E (POLR3E)	
RNA polymerase III subunit H (POLR3H)	
TWIST neighbor (TWISTNB)	
DEAD-box helicase 5 (DDX5)	
PHD finger protein 5A (PHF5A)	
RNA binding motif protein, X-linked (RBMX)	
elongation factor Tu GTP binding domain containing 2 (EFTUD2)	
heterogeneous nuclear ribonucleoprotein M (HNRNPM)	
peptidylprolyl isomerase E (PPIE)	
peptidylprolyl isomerase H (PPIH)	
peptidylprolyl isomerase like 1 (PPIL1)	
pre-mRNA processing factor 4 (PRPF4)	
small nuclear ribonucleoprotein polypeptide A’(SNRPA1)	
small nuclear ribonucleoprotein polypeptide A (SNRPA)	
small nuclear ribonucleoprotein polypeptide B2 (SNRPB2)	
small nuclear ribonucleoprotein polypeptide E (SNRPE)	
small nuclear ribonucleoprotein polypeptide F (SNRPF)	
small nuclear ribonucleoprotein polypeptides B and B1 (SNRPB)	
splicing factor 3a subunit 2 (SF3A2)	

### Cluster III: genes upregulated 4 to 13 weeks after spawning

Cluster III comprised approximately 300 unique genes. DAVID analysis of the eligible genes showed enrichment of this cluster with genes related to translation, most of which encode ribosomal subunits. Cluster III was also enriched with genes involved in protein folding mediated by the chaperonin-containing-T-complex. Of note, cluster III contained myogenin the only myogenic regulatory factor found to be upregulated during the post-spawning period. Overall, cluster III was dominated by genes regulating protein biosynthesis and maturation for cell growth (for details see Tables 1 and 6; the list of genes that formed the major functional categories of cluster III are in additional file 4).

**Table 6:** Genes upregulated 4 to 13 weeks after spawning (cluster III) and involved in ribosome

Genes from cluste III involved in ribosome	
60S ribosomal protein L37 (RPL37)	
ribosomal protein L10 (RPL10)	
ribosomal protein L10a (RPL10A)	
ribosomal protein L11 (RPL11)	
ribosomal protein L12 (RPL12)	
ribosomal protein L13 (RPL13)	
ribosomal protein L13a (RPL13A)	
ribosomal protein L14 (RPL14)	
ribosomal protein L15 (RPL15)	
ribosomal protein L18 (RPL18)	
ribosomal protein L18a (RPL18A)	
ribosomal protein L19 (RPL19)	
ribosomal protein L21 (RPL21)	
ribosomal protein L22 (RPL22)	
ribosomal protein L23 (RPL23)	
ribosomal protein L24 (RPL24)	
ribosomal protein L27 (RPL27)	
ribosomal protein L27a (RPL27A)	
ribosomal protein L29 (RPL29)	
ribosomal protein L3 like (RPL3L)	
ribosomal protein L3 (RPL3)	
ribosomal protein L30 (RPL30)	
ribosomal protein L31 (RPL31)	
ribosomal protein L32 (RPL32)	
ribosomal protein L34 (RPL34)	
ribosomal protein L34 (RPL34)	
ribosomal protein L35 (RPL35)	
ribosomal protein L35a (RPL35A)	
ribosomal protein L39 (RPL39)	
ribosomal protein L4 (RPL4)	
ribosomal protein L5 (RPL5)	
ribosomal protein L6 (RPL6)	
ribosomal protein L7a (RPL7A)	
ribosomal protein L8 (RPL8)	
ribosomal protein L9 (RPL9)	
ribosomal protein S10 (RPS10)	
ribosomal protein S11 (RPS11)	
ribosomal protein S12 (RPS12)	
ribosomal protein S13 (RPS13)	
ribosomal protein S14 (RPS14)	
ribosomal protein S15 (RPS15)	
ribosomal protein S15a (RPS15A)	
ribosomal protein S16 (RPS16)	
ribosomal protein S17 (RPS17)	
ribosomal protein S18 (RPS18)	
ribosomal protein S19 (RPS19)	
ribosomal protein S2 (RPS2)	
ribosomal protein S20 (RPS20)	
ribosomal protein S23 (RPS23)	
ribosomal protein S26 (RPS26)	
ribosomal protein S27 (RPS27)	
ribosomal protein S3 (RPS3)	
ribosomal protein S3A(RPS3A)	
ribosomal protein S4, X-linked (RPS4X)	
ribosomal protein S5 (RPS5)	
ribosomal protein S6 (RPS6)	
ribosomal protein S8 (RPS8)	
ribosomal protein S9 (RPS9)	
ribosomal protein SA (RPSA)	
ribosomal protein lateral stalk subunit P0 (RPLP0)	
ribosomal protein lateral stalk subunit P1 (RPLP1)	
ribosomal protein lateral stalk subunit P2 (RPLP2)	

### Cluster IV: genes upregulated 8 to 24 weeks after spawning

Cluster IV contained approximately 940 unique genes. In agreement with the downregulation of genes involved in aerobic ATP production after spawning, cluster IV was highly enriched with genes involved in glycolysis. Cluster IV was also enriched with genes regulating amino acid biosynthesis and genes involved in the formation of extracellular matrix or encoding components of sarcomeres such as myosins and troponins. Finally, cluster IV contained many genes involved in cell proliferation and division. Overall, cluster IV was dominated by genes regulating glycolysis, cell cycle-related genes and genes encoding structural components of myofibres (for details see Tables 1 and 7; the list of genes that formed the major functional categories of cluster IV are in Additional file 5).

**Table 7:** Genes upregulated 8 to 24 weeks after spawning (cluster IV) and involved in glycolysis/ neoglucogenesis

Genes from cluster IV involved in glycolyse/neoglucogenesis	
aldehyde dehydrogenase 2 family (mitochondrial) (ALDH2)	
aldolase, fructose-bisphosphate A (ALDOA)	
aldolase, fructose-bisphosphate B (ALDOB)	
aldolase, fructose-bisphosphate C (ALDOC)	
enolase 1 (ENO1)	
enolase 2 (ENO2)	
enolase 3 (ENO3)	
fructose-bisphosphatase 1 (FBP1)	
fructose-bisphosphatase 2 (FBP2)	
glucose-6-phosphate isomerase (GPI)	
glyceraldehyde-3-phosphate dehydrogenase (GAPDH)	
hexokinase 1 (HK1)	
lactate dehydrogenase A (LDHA)	
lactate dehydrogenase B (LDHB)	
phosphofructokinase, liver type (PFKL)	
phosphofructokinase, muscle (PFKM)	
phosphofructokinase, platelet (PFKP)	
phosphoglucomutase 1 (PGM1)	
phosphoglycerate kinase 1 (PGK1)	
phosphoglycerate kinase 2 (PGK2)	
phosphoglycerate mutase 1 (PGAM1)	
phosphoglycerate mutase 2 (PGAM2)	
pyruvate kinase, muscle (PKM)	
triosephosphate isomerase 1(TPI1)	

### A specific muscle transcriptional programme is associated with post-spawning fillet quality recovery

**Fig. 2:**
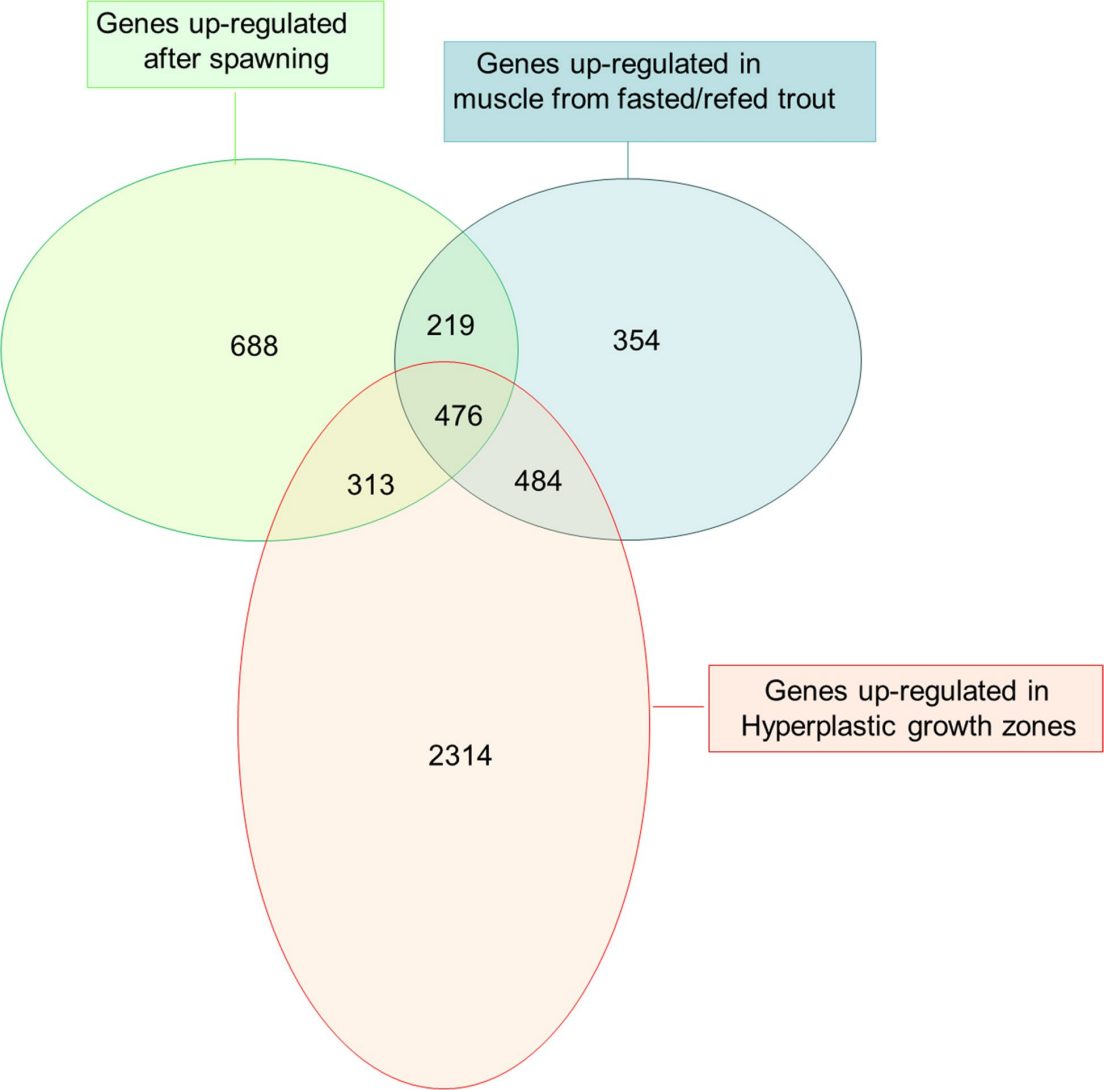
Venn diagram representing the distribution of genes upregulated in muscle during post spawning recovery, after refeeding following fasting and in the superficial hyperplastic growth zones of the myotome in late trout embryos. Approximately 700 genes were found to be specifically upregulated during post-spawning muscle recovery

To further characterize the specificity of the transcriptional programme associated with fillet yield and flesh quality recovery after spawning, we compared it (i.e. that of clusters II + III + IV) with that induced by a fasting-refeeding schedule [13] and that associated with the hyperplastic growth area of the late trout embryo myotome as identified using laser capture microdissection and microarray analysis [12]. A Venn diagram (Fig. 2) showed that the transcriptional programme associated with fillet yield and flesh quality recovery included approximately 700 specific overexpressed genes that were not found to be upregulated in muscle from fasted/refed trout or in hyperplastic growth zones. DAVID analysis showed that the most enriched functional categories for the genes that were specifically upregulated after spawning were related mainly to ribosomal proteins and glycolysis. Interestingly, many genes found to be overexpressed in hyperplastic growth zones and involved in myofiber production [12], notably, genes encoding canonical myogenic transcriptional regulators such as Pax3, Pax7, MyoD1a, MyoD1b, myf5 and mrf4 and genes encoding membrane receptors regulating myogenic cell fusion such as M-cadherin, Brother of CDO, protogenin, Jamb and Kin of Irre-like 3, were not upregulated after spawning. Additionally, most of the myosins and tropomyosins specific to nascent myofibres that form in hyperplastic growth zones of the prehatching trout myotome were not found to be overexpressed after spawning. Overall, a muscle transcriptional programme promoting anaerobic ATP production, myofibre hypertrophic growth and extracellular matrix remodelling, but not new myofibre formation, was associated with post-spawning fillet quality recovery.

## Discussion

Sexual maturation causes loss of fish muscle mass and deterioration of fillet quality attributes that prevent market success. We recently showed that fillet yield and flesh quality in mature female trout that have spawned can evolve to become similar to those of control immature female trout [11]. In this study, to gain insights into the molecular mechanisms regulating fillet quality recovery, we examined the evolution of the muscle transcriptome in female trout after spawning. Consistent with post-spawning flesh quality recovery, we observed that the muscle transcriptome after spawning evolved to eventually become similar to that of trout that did not experience spawning. In keeping with this evolution, the muscle transcriptome after spawning exhibited gradual downregulation (cluster I) of a large set of genes previously shown to be overexpressed in the muscle tissues of mature fertile female trout compared to those of immature and/or triploid sterile female trout [7–9, 14]. Specifically, we observed sharp decreases in the expression of genes involved in muscle proteolysis and especially in the expression of genes regulating the ubiquitin-proteasome pathway, involved in autophagy or encoding lysosomal proteases. This suggests that a decrease in protein breakdown is essential for muscle mass gain and quality recovery after spawning. Additionally, we observed downregulation of genes involved in mitochondrial energetic metabolism, such as genes of the TCA cycle, the respiratory chain and mitochondrial ATP synthesis. Conversely, genes involved in the cytosolic glycolysis pathway were upregulated during the post-spawning recovery period (cluster IV). In line with this shift in energy metabolism to become increasingly anaerobic, we also observed decreased abundance of transcripts involved in mitochondrial fatty acid oxidation (cluster I), a pathway that fuels aerobic ATP production. Surprisingly, only very few genes involved in the biosynthesis of fatty acids were found to be upregulated in muscle after spawning. This finding, however, is in line with studies reporting that endogenous lipids are synthesised mainly in the liver before being transported to peripheral tissues such as muscle [15].

We have previously reported that muscle firmness decreases during the post-spawning period [11]. Our transcriptomic analysis showing parallel downregulation (cluster I) of genes encoding proteasome components and genes encoding intracellular proteases such as lysosomal cathepsins is in line with a previous report that positively correlated salmon flesh firmness and the expression of genes belonging to these functional categories [16]. However, this finding contrasts with previous works reporting higher rates of protein degradation in fillets with low firmness than in fillets with high firmness [6, 17–19]. With regards to these discrepancies, one must keep in mind that firmness results from multifactorial interactions [20]. It is likely that, in agreement with many studies documenting softer flesh in fattier fish [21–23], fat accumulation occurring after spawning [11] contributes to the increase in flesh softness observed during this period. To explain the effects of adiposity on flesh firmness, it has been proposed that muscle fibres embedded with large amounts of fat easily slide across each other and hence offer less force of resistance to compression [24]. In addition, it is worth mentioning that the lipid content of the muscle is also thought to influence flavour and “juiciness” , both of which are major traits of flesh quality in fish [19, 25].

A striking feature of the transcriptomic signature following spawning is the upregulation (cluster IV) of a large set of genes encoding structural components such as extracellular matrix proteins that form the intricate matrix network surrounding individual myofibres and blocks of myofibres and sarcomeric proteins that assemble to generate contractile myofilaments. The overexpression of extracellular matrix proteins during the post-spawning period is likely to contribute to muscle structure rebuilding and concurrent flesh quality recovery. In keeping with this latter point, the amount and composition of the extracellular matrix have been reported to be determinants of textural quality [20]. Thus, some studies have reported a positive association between fillet firmness and collagen content [26–29]. In contrast to these studies, but in agreement with those by Moreno et al. [30] and Larsson et al. [16], we did not observe in our study that extracellular matrix component overexpression was associated with flesh firmness. However, it is important to point out that not only the amounts of extracellular matrix components but also the levels of cross-linkage between them impact textural properties [31, 32].

The overexpression of contractile protein-encoding genes (cluster IV) and the enrichment in functional categories related to protein biosynthesis and maturation (cluster II) suggest that accretion of protein mass occurs in muscle fibres after spawning. Further supporting the view of hypertrophic growth of muscle fibres, we also found (cluster II) strong enrichment of genes stimulating ribosome biogenesis, a crucial mechanism used by skeletal muscle to regulate protein synthesis and control muscle mass [33]. Our observation showing that post-spawning hypertrophic growth is associated with a decrease in flesh firmness is consistent with the findings of previous studies establishing a relationship between muscle fibre density and firmness [20]. Interestingly, most functional categories inferred for genes upregulated in muscle after spawning and related to muscle hypertrophic growth have also been reported to be activated in muscle from refed trout after 1 month of fasting [13, 34]. However, the number of differentially expressed genes after spawning is more important than that found after refeeding. This finding suggests that muscle damage induced by sexual maturation and egg production is more important than that provoked by fasting and that its reversal involves a more drastic transcriptional response. Regardless, during the post-spawning period, as in the course of a fasting/refeeding schedule [13], most of the genes regulating hyperplastic growth or encoding contractile proteins specific to nascent trout myofibres were not overexpressed. This suggests that the production of new myofibres in trout adulthood is not stimulated during muscle remodelling following muscle mass loss. This feature contrasts with the situation in trout muscle regeneration, during which a large part of the transcriptional programme underlying muscle hyperplasia is reactivated [35]. An in situ visualization of differentiating myocytes expressing myomaker and/or myomixer, two essential muscle-specific fusion proteins recently discovered in vertebrates, including fish [36], would definitively confirm the absence of hyperplastic growth resumption after spawning. Cell cycle-related genes were found to be upregulated after spawning (Cluster IV). It is then tempting to speculate that myogenic progenitors proliferate after spawning to enable myonuclear accretion necessary for muscle fibre hypertrophy. In keeping with this point, it is interesting to note that myogenin, a myogenic factor regulating vertebrate myogenic differentiation, has recently been shown to be essential for myonuclear accretion and proper muscle fibre growth in fish [37]. Our observation that myogenin (and not others myogenic regulatory factors such as Myod1a, Myod1b, myf5 and mrf4) was transiently upregulated after spawning could be related to a specific role of myogenin in post-spawning muscle fibre hypertrophy.

## Conclusion

In this study, we show that the recovery of fillet yield and flesh quality that follows trout spawning is associated mainly with dynamic transcriptional changes promoting anaerobic ATP production, muscle fibre hypertrophic growth and extracellular matrix remodelling. Many genes from the post-spawning transcriptional signature are potentially important determinants for fish muscle growth and/or flesh quality. As such, they deserve further expression and functional analyses and could be assessed for use in marker-assisted selection of trout with superior muscle yield and fillet quality traits.

## Methods

### Fish sampling and experimental design

The fish used in this study have been previously described [11]. Diploid female rainbow trout *(Oncorhynchus mykiss)* from the same autumnal strain cohort were reared in the INRAE’s experimental facilities (PEIMA, Sizun) France). After ovulation and stripping, females that spawned on the same date were placed into a circular 2-m-diameter tank randomly chosen containing 2 m^3^ of water. A total of nine experimental groups of post-spawning fish were created. The fish were fed the same diet throughout the course of the trial. During sampling, post-spawning fish (*n* = 20) from the same tank were sequentially slaughtered at 0, 1, 2, 4, 8, 13, 16, 24, and 33 weeks after ovulation. Immature (control) female trout (*n* = 20) belonging to the same cohort as trout that experienced spawning were also sampled at the beginning (C0) and at the end (C33) of the experiment. At slaughter, the fish were anaesthetized with Tricaine Pharmaq (5 g/100 L) in a 500 L tank, killed by a blow to the head, and then bled by gill cutting. After death, quality parameters of the fish were measured, and a slice of white skeletal muscle was carefully dissected from the dorsal region of the musculature, frozen in liquid nitrogen and stored at − 80 °C until RNA extraction. Muscle tissues of eight trout at different post-spawning (PS) timepoints (PS0, PS2, PS4, PS8, PS13, PS16, PS24, PS33) as well as muscle tissues of eight control (immature) trout (C0 and C33) were subjected to RNA extraction and transcriptome analysis. The fish used for transcriptome analyses were selected on the basis of their carcass weight which had to be similar to the median value of the group to which they belonged.

### RNA extraction, labelled cRNA generation and hybridization

Total RNA extraction was performed using TRIzol reagent (Invitrogen, Carlsbad, CA, USA) reagent following the manufacturer’s instructions. RNA integrity was assessed with an Agilent 2100 Bioanalyzer. Cy3-labelled cRNA generation and hybridization were performed as previously described [12]. Hybridizations were carried out using Agilent 8x60K high-density oligonucleotide microarray slides (GEO platform record: GPL15840) [12].

### Data acquisition and analysis

Hybridized slides were rinsed and scanned at a 3-μm thickness with an Agilent DNA Microarray Scanner. Fluorescence intensity was calculated using the standard procedures found in Agilent Feature Extraction (FE) software 10.7.3.1. The arrays were normalized and log-transformed using GeneSpring software (version 14.9). An unpaired t test (Benjamini-Hochberg-corrected *p*-val < 0.05) was used to specifically compare the muscle transcriptome of trout that had just spawned (PS0) with that of control trout (C0) and to compare the muscle transcriptome of trout at 33 weeks post-spawning (PS33) with that of 33 week control trout (C33). A one-way ANOVA (Benjamini-Hochberg method with an FDR < 0.05) and a fold change > 3 were used as the criteria for defining genes whose expression levels were significantly different across all the samples (i.e. PS0, PS2, PS4, PS8, PS13, PS16, PS24, PS33) examined. For clustering analysis, the data were median-centred and average linkage clustering was carried out using CLUSTER software (version 3.0). The clusters were visualized with TreeView (version 1.1.6r4) [38]. GO enrichment analysis of the DEG list from each cluster was performed using the Database for Annotation, Visualization and Integrated Discovery (DAVID, 6.8) software tools [39, 40].

## Supplementary Information

**Additional file 1.**

**Additional file 2.**

**Additional file 3.**

**Additional file 4.**

**Additional file 5.**

## Data Availability

Gene expression data supporting this article are available in the Genexpression Omnibus (GEO) repository under the accession number: GSE165933: https://www.ncbi.nlm.nih.gov/search/all/?term=GSE165933
